# Efficacy and safety of 5-hydroxytryptamine 3 receptor antagonists in irritable bowel syndrome: A systematic review and meta-analysis of randomized controlled trials

**DOI:** 10.1371/journal.pone.0172846

**Published:** 2017-03-14

**Authors:** Yongping Zheng, Ting Yu, Yurong Tang, Wenjie Xiong, Xiaoxue Shen, Ling Jiang, Lin Lin

**Affiliations:** Department of Gastroenterology, First Affiliated Hospital of Nanjing Medical University, Nanjing, Jiangsu Province, China; University Hospital Llandough, UNITED KINGDOM

## Abstract

**Aim:**

We assessed the efficacy and safety of 5-hydroxytryptamine (5-HT_3_) receptor antagonists in adults with non-constipated irritable bowel syndrome (IBS) or diarrhea-predominant IBS (IBS-D).

**Methods:**

We searched PubMed, MEDLINE, EMBASE, and the Cochrane Controlled Trials Register for randomized controlled trials (RCTs) involving adults with non-constipated IBS or IBS-D that compared 5-HT_3_ receptor antagonists with placebo or other conventional treatment. Dichotomous symptom data were pooled to obtain the relative risk (RR) and 95% confidence intervals (CIs) for improving global IBS symptoms, abdominal pain and abnormal bowel habits, or stool consistency symptoms after therapy, and adverse events, including constipation. Meta- analysis was performed with Mantel Haenszel method using Revman 5.3 software.

**Results:**

We included 21 RCTs; 16 were high quality (Jadad score ≥ 4). The pooled RR of global IBS symptoms improved by 5-HT_3_ receptor antagonists versus placebo or mebeverine was 1.56 (95% CI: 1.43–1.71); alosetron, ramosetron, and cilansetron had similar treatment effects. The pooled RR of abdominal pain relieved by 5-HT_3_ receptor antagonists versus placebo was 1.33 (95% CI: 1.26–1.39). The pooled RR showed that 5-HT_3_ receptor antagonists improved abnormal bowel habits or stool consistency symptoms (RR = 1.63, 95% CI: 1.33, 1.99). The pooled RR of adverse events following 5-HT_3_ receptor antagonist treatment was 1.15 (95% CI: 1.08, 1.22). Subgroup analysis indicated that alosetron had a high rate of adverse effects (RR = 1.16, 95% CI: 1.08, 1.25); adverse events following ramosetron treatment were not statistically significantly different. 5-HT_3_ receptor antagonists were likelier to cause constipation: the pooled RR of constipation developing with 5-HT_3_ receptor antagonist versus placebo was 3.71 (95% CI: 2.98–4.61). However, constipation was likelier in patients with non-constipated IBS after taking 5-HT_3_ receptor antagonists than in patients with IBS-D only (non-constipated IBS and IBS-D: RR = 5.28 [95% CI: 3.93, 7.08] vs. IBS-D only 3.24 [2.54, 4.12]).

**Conclusions:**

Ramosetron, cilansetron, ondansetron, and alosetron are effective for treating non-constipated IBS and IBS-D. Our systematic review found rare serious adverse events.

## Introduction

Irritable bowel syndrome (IBS) is one of the most common functional bowel diseases, and is characterized by abdominal pain and abnormal bowel habits [1]. According to the Rome III criteria, IBS is classified into four subtypes: with diarrhea (IBS-D), with constipation (IBS-C), mixed type (IBS-M), and unsubtyped (IBS-U) [2]. Understanding of the pathogenesis of IBS remains incomplete. The mechanism of its etiology may be related to altered bowel motility and visceral hypersensitivity [3]. Its effect on society is now well-recognized because the quality of life of individuals with IBS is profoundly disrupted, and it causes economic loss to such individuals and society due to the medical consultations required and the consumption of other valuable resources. Nonetheless, treatments for IBS are unsatisfactory; only 22% of patients with IBS receiving conventional medical care report at least 50% reduction in bowel symptoms [4].

Many agents have been developed for treating IBS-D. 5-Hydroxytryptamine (5-HT) receptors are classified into seven subtypes, and 5-HT3 receptors are known to be localized on intestinal plexuses, sensory nerves, sympathetic and parasympathetic nerves, to stimulate the release of neurotransmitters. 5-HT acts on the 5-HT_3_ receptors on the parasympathetic ganglia to cause smooth muscle contraction and increased intestinal secretion by stimulating nerve terminal acetylcholine release [5]. 5-HT_3_ receptor antagonists inhibit the activation of 5-HT_3_ receptors on the mucosal processes of the intrinsic and extrinsic primary afferent neurons and attenuate motor and secretory reflex activity while decreasing the depolarization of extrinsic sensory neurons that transmit signals to the brain, thereby inhibiting the sensory signals leading to abdominal pain and discomfort and are likely directly or indirectly related to the pathophysiology of IBS [6]. This has been exploited therapeutically in patients with IBS-D in whom 5-HT_3_ receptor antagonists improve gastrointestinal (GI) symptoms, reducing stool frequency, urgency, and abdominal discomfort while increasing stool consistency [7]. In patients with IBS-D, 5-HT_3_ receptor antagonists also improve IBSQOL, treatment satisfaction, daily activities, and lost workplace productivity (LWP) [8]. A novel selective 5-HT_3_ receptor antagonist, ramosetron, was initially used in Japan in animal experiments involving stress-induced defecation disturbance and inhibitory effects on colonic nociception [9]. Several recent clinical trials confirmed that ramosetron can be used for patients with IBS-D. No serious ramosetron-related adverse event, specifically ischemic colitis, has been observed in patients with either dose of ramosetron [10]. However, these findings may not be generalizable to Western populations. Alosetron is a selective 5-HT_3_ receptor antagonist that significantly improves abnormal bowel function and relieves pain and discomfort in IBS-D [8]. Unfortunately, a minority of patients treated subsequently with alosetron experienced serious adverse events, and alosetron was voluntarily withdrawn due to post-marketing reports of ischemic colitis and the complication of constipation [11]. Other 5-HT_3_ receptor antagonists include ondansetron and cilansetron. Ondansetron inhibits the nausea and vomiting associated with chemotherapy. A recent study discovered that compared with placebo, patients given ondansetron experienced fewer days with urgency, lower urgency scores, reduced frequency of defecation, and less bloating [12]. These effects developed rapidly within 7 days; <10% of patients had constipation, and there were no cases of ischemic colitis. These findings have important implications for clinicians, as ondansetron is an inexpensive drug with a good safety profile [13]. Three randomized controlled trials (RCTs) found that cilansetron was more effective than placebo at improving the overall symptoms of IBS-D, including abdominal pain and diarrhea, in female and male patients. The most commonly reported adverse effect is constipation, and the drug has generally been well-tolerated in clinical trials. Although rare, the adverse effect of greatest concern is suspected ischemic colitis, similar to alosetron. How the issues around cilansetron safety will affect the approval process in various countries remains to be determined. A detailed risk management plan and post-marketing surveillance program will be required should this drug become available for treating IBS-D [14].

Previous systematic reviews and meta-analyses that examined alosetron and cilansetron efficacy for treating IBS either have important limitations or are outdated [4,15]. In addition, no single existing systematic review and meta-analysis has synthesized the current available evidence to examine the efficacy of all 5-HT_3_ receptor antagonists in patients with IBS, nor has any systematic review and meta-analysis examined the efficacy and adverse events of all available 5-HT_3_ receptor antagonists in IBS through synthesis of the current available evidence. Such information would be important both for developing newer pharmaceutical agents that act on the 5-HT_3_ receptor and transport system and for understanding the role of these agents for treating IBS. More importantly, several new studies on 5-HT_3_ receptor antagonist treatment of IBS have been published in recent years. Accordingly, we conducted this systematic review and meta-analysis to examine the efficacy of the available 5-HT_3_ receptor antagonists for treating IBS, and performed subgroup analyses according to treatment, sex, dose and duration of therapy, study population, publication status, and publication year to obtain more accurate and comprehensive results regarding the efficacy and adverse effects of 5-HT_3_ receptor antagonists in IBS treatment.

## Methods

This meta-analysis was conducted according to the Preferred Reporting Items for Systematic Reviews and Meta-Analyses guidelines (PRISMA) 20 (S1 Text). We searched the medical literature in PubMed, MEDLINE (1950 to July 2016), EMBASE (1980 to July 2016), and the Cochrane Controlled Trials Register (2016). The following terms were used to identify IBS: functional gastrointestinal disorder, refractory irritable bowel symptoms, irritable bowel syndrome, non-constipated irritable bowel syndrome, non-constipated IBS, diarrhea-predominant irritable bowel syndrome, IBS-D, IBS. These terms were combined using the set operator AND with studies identified with the following terms: serotonin antagonists, receptors (serotonin, 5-HT_3_) (both as MeSH and free text terms), and the following free text terms: 5-HT_3_, ramosetron, alosetron, cilansetron, ondansetron.

### Inclusion and exclusion criteria

Inclusion criteria: (1) RCTs. (2) Adults (aged >16 years) diagnosed with IBS based on clinician opinion or having met diagnostic Rome I, II, or III criteria; negative investigations were used as a supplement. (3) 5-HT_3_ receptor antagonists were compared with placebo or conventional therapy. (4) Minimum 1-week therapy duration. (5) Abdominal pain or IBS symptoms global assessment following therapy. (6) Abnormal bowel habits or stool consistency symptoms following therapy.

Exclusion criteria: (1) IBS not distinguished from functional GI disorder. (2) Age < 18 years. (3) No 5-HT_3_ receptor antagonist treatment groups or combined 5-HT_3_ receptor antagonists for a single patient. (4) Data from original literature could not be extracted. (5) First period outcome data not provided by cross-over studies. (6) Duplicate publication. (7) Language was not English.

### Outcome assessment

We assessed the primary outcomes 5-HT_3_ receptor antagonist effect on abdominal pain or global IBS symptoms as compared with placebo or mebeverine. A study designed proposed by the US Food and Drug Administration (FDA) for clinical trials focused on IBS suggested the use of stool consistency as a co-primary endpoint for IBS-D [16]. Accordingly, our secondary outcomes were 5-HT_3_ receptor antagonist effects on stool consistency–related symptoms or abnormal bowel habits and common adverse events, including constipation. We also analyzed the efficacy by IBS type according to drug, gender, dose, therapy duration, study population, publication type, and publication year.

### Literature quality evaluation

The Jadad score, which analyzes the quality of a research article based on random sequence generation, randomization concealment, blinding, and dropouts, was used to evaluate eligible article quality. Low-quality articles have a Jadad score between 1 and 3; high-quality articles have a Jadad score between 4 and 7.

### Data extraction

Two reviewers extracted all data independently to a Microsoft Excel spreadsheet as dichotomous outcomes (improvement in global IBS symptoms, abdominal pain, or abnormal bowel habits or stool consistency). We also extracted the following clinical data for each RCT: country of origin, 5-HT_3_ receptor antagonist dose, therapy duration, total adverse events reported, IBS definition criteria, outcome measure for defining symptom improvement or cure following therapy, proportion of female patients. Where the trial reporting allowed, we extracted the data as intention-to-treat analyses, where all dropouts were assumed to be treatment failures.

### Data synthesis and statistical analysis

We used Review Manager (RevMan) 5.3 (RevMan for Windows 2010, the Nordic Cochrane Centre, Copenhagen, Denmark) to calculate the pooled effect size and used Stata 12.0 to assess publication bias and sensitivity analysis. We pooled data using Mantel-Haenszel hybrid with inverse variance weighting to allow for random effects model to yield more conservative estimates of the effects of individual treatments. The intervention effects are expressed as relative risk (RR) with 95% confidence intervals (CIs) of abdominal pain, global IBS symptoms, and abnormal bowel habits or stool consistency improving with intervention.

Individual study results can be diverse; a single meta-analysis can quantify this inconsistency with a statistical test of heterogeneity to determine whether the variation across trials stems from true heterogeneity or from chance. The quantity, *I*^*2*^, ranges 0–100%: We used *I*^*2*^ < 25% to represent low heterogeneity; effect size was pooled using a fixed effect model. *I*^*2*^ > 25% and <50% suggested moderate heterogeneity; *I*^*2*^ > 50% indicated significant heterogeneity [17]. Taking a conservative approach, we used a random effects model, which produces wider CIs than a fixed effect model [18]. We conducted sensitivity analysis when there was significant heterogeneity, which allowed us to find the source thereof and to evaluate the robustness of the results. Subgroups were analyzed based on study population (non-constipated IBS vs. IBS-D only), sex (female vs. male or mixed), 5-HT_3_ receptor antagonist, treatment duration (4–12 weeks vs. 24–48 weeks, i.e., long-term), and publication status (abstract only vs. full-text).

We used RevMan 5.3 to generate forest plots of pooled RRs and risk differences with 95% CIs for primary and secondary outcomes, and generated funnel plots. We used the Begg and Egger tests to assess the funnel plots for asymmetry and therefore possible publication bias when included trails were >10 [19].

## Results

**Fig 1:**
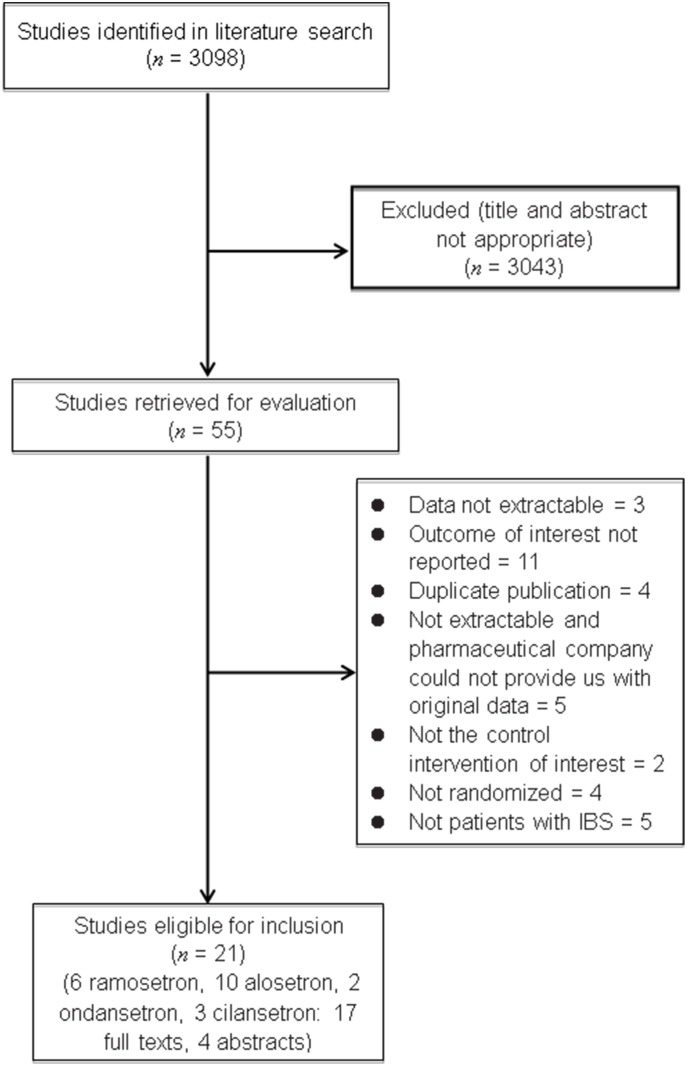
Detailed screening process.

Our search returned an initial 3099 citations, of which 55 appeared relevant to the systematic review and were retrieved for assessment. Based on the inclusion and exclusion criteria, 21 RCTs were eligible for inclusion. Fig 1 depicts the detailed screening process.

The 21 RCTs involved 10,898 patients with IBS defined according to the Rome criteria: six investigated ramosetron [9, 20–24], 10 assessed alosetron [25–34], and two compared ondansetron [12, 35] with placebo or mebeverine; these studies all scored ≥4 on the Jadad scale. However, the three cilansetron studies had Jadad scores of 3 [36–38]. Table 1 details the RCT characteristics.

**Table 1:** Characteristics of randomized controlled trials of 5-HT_3_ receptor antagonists versus placebo in IBS.

Study (first author, year, reference no.)	Country	Diagnostic criteria	IBS type	Defining criteria for symptom improvement following therapy	Sex	Sample size	Drop out	Adverse events	Treatment	Duration	Jadad score
Ramosetron											
Matsueda 2008^22^	Japan	Rome II	IBS-D	Improvement of overall IBS symptoms; improvement of abdominal pain/discomfort; improvement of abnormal bowel habits or stool consistency	Mixed	418	17/13/17/21	55/60/62/61	1 μg once daily; 5 μg once daily; 10 μg once daily, orally	12 weeks	6
Matsueda 2008^23^	Japan	Rome II	IBS-D	Improvement of overall IBS symptoms; improvement of abdominal pain/discomfort; improvement of abnormal bowel habits or stool consistency	Mixed	539	41/46	163/141	5 μg once daily, orally	12 weeks	5
Lee 2011^21^	Korea	Rome III	IBS-D	Improvement of overall IBS symptoms; improvement of abdominal pain/discomfort; improvement of abnormal bowel habits/stool consistency	Male	343	13/15	13/06/00	5 μg once daily; 135 mg mebeverine three times daily, orally	4 weeks	3
Fukudo 2014^20^	Japan	Rome III	IBS-D	Improvement of overall IBS symptoms; improvement of abdominal pain/discomfort; improvement of abnormal bowel habits/stool consistency	Male	296	10/18/00	69/77	5 μg once daily, orally	12 weeks	7
Fukudo 2015^19^	Japan	Rome III	IBS-D	Improvement of overall IBS symptoms	Female	409	Unclear	Unclear	1.25 μg once daily; 2.5 μg once daily; 5 μg once daily, orally	12 weeks	3
Fukudo 2016^4^	Japan	Rome III	IBS-D	Improvement of overall IBS symptoms; improvement of abdominal pain/discomfort; improvement of abnormal bowel habits/stool consistency	Female	576	26/20	154/118	2.5 μg once daily, orally	12 weeks	7
Ondansetron											
Maxton 1996^34^	UK	Rome criteria	IBS	Improvement of abdominal pain/discomfort; improvement of abnormal bowel habits/stool consistency	Mixed	50	1	Unclear	4 mg orally three times daily	4 weeks	4
Garsed 2014^12^	UK	Rome III	IBS-D	Improvement of abnormal bowel habits/stool consistency	Mixed	120	22	7/6	4 mg, orally, two tablets three times daily crossover study of ondansetron 4 mg/tablet versus placebo	8 weeks	7
Alosetron											
Jones 1999^33^	UK	Rome I	Non-constipated IBS	Adequate relief of abdominal pain and discomfort	Female	628	68/58	220/196	1 mg twice daily; 135 mg mebeverine three times daily, orally	12 weeks	6
Camilleri 1999^32^	USA	Rome criteria	Non-constipated IBS	Adequate relief of pain and discomfort for at least 6 of the 12 weeks of therapy	Mixed	370	15, 22, 20, 20/12	Unclear	1, 2, 4, or 8 mg twice daily, orally	12 weeks	4
Camilleri 2000^31^	USA	Rome criteria	IBS-D	Improvement of abdominal pain/discomfort	Female	647	79/53	233/210	1 mg orally twice daily	12 weeks	7
Bardhan 2000^30^	UK	Rome I	29% IBS-D, 71% non-constipated IBS	Improvement in abdominal pain/discomfort (on visual analog scale), abnormal bowel habits or stool consistency	Mixed	462	33, 41, 32/33	56, 63, 64/60	0.1 mg twice daily, 0.5 mg twice daily, and 2 mg twice daily, orally	12 weeks	6
Camilleri 2001^28^	USA	Rome I	71% IBS-D, 29% non-constipated IBS	Adequate relief of IBS pain and discomfort for at least 2 weeks per month	Female	626	72/71	118/62	1 mg orally twice daily	12 weeks	7
Lembo 2001^29^	USA	Rome II	IBS-D	Improvement of overall IBS symptoms	Female	801	99/50	409/178	1 mg twice daily, orally	12 weeks	4
Lembo 2004^26^	USA	Rome II	IBS-D	Percentage of days with satisfactory control of bowel urgency, response rate for the IBS global improvement scale (GIS)	Female	492	99/50	145/127	1 mg twice daily, orally	12 weeks	4
Chey 2004^27^	USA	Rome I	80% IBS-D, 20% more frequent urgency	Weekly adequate relief of IBS pain and discomfort at week 48 of treatment; improvement of abnormal bowel habits or stool consistency	Female	714	Unclear	297/261	1 mg twice daily, orally	48 weeks	4
Changl 2005^25^	USA	Rome I	IBS-D	Average adequate relief of IBS pain and discomfort for last 8 weeks of treatment	Male	662	22, 24, 31, 21/18	73, 86, 80, 87/65	0.5, 1, 2, or 4 mg twice daily, orally	12 weeks	6
Krause 2007^24^	USA	Rome II	IBS-D	Improvement of overall IBS symptoms; improvement of abdominal pain/discomfort; improvement of abnormal bowel habits or stool consistency	Female	705	60/74/85/65	107/101/102/94	0.5 mg once daily, 1 mg once daily, 1 mg twice daily, orally	12 weeks	7
Cilansetron											
Bradette 2004^35^	Unspecified	Unspecified	IBS-D	Adequate relief of IBS symptoms in ≥50% of weekly diary responses	Mixed	792	Unclear	Unclear	2 mg three times daily, orally	6 months	3
Francisconi 2005^36^	Unclear	Unspecified	IBS-D	IBS symptoms interfered rarely or not at all with activities over the past 4 weeks	Unclear	746	Unclear	Unclear	2 mg three times daily, orally	12 weeks	3
Miner 2004^37^	USA	Unspecified	IBS-D	Adequate relief of IBS symptoms in ≥50% of weekly diary responses	Unclear	692	Unclear	Unclear	2 mg three times daily, orally	3 months	3

### Efficacy of 5-HT_3_ receptor antagonists in the treatment of IBS

**Fig 2:**
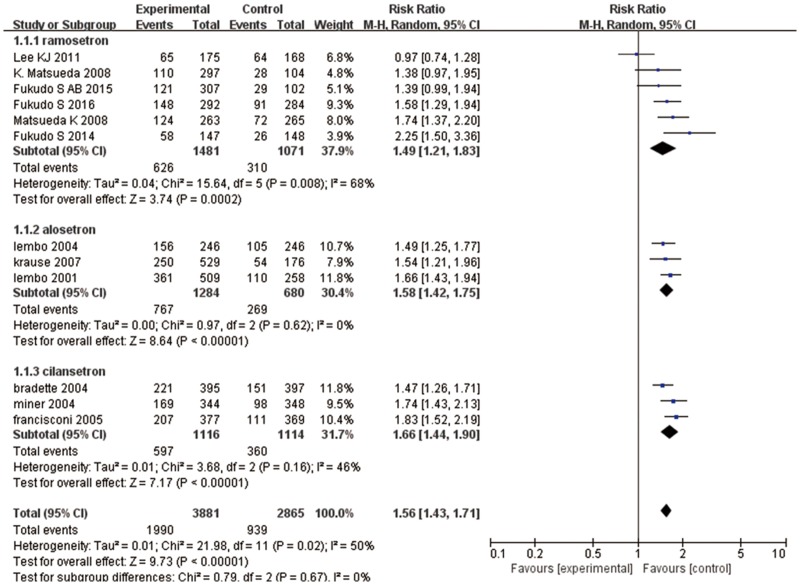
Forest plot of the improvement in global IBS symptom improvement. Twelve articles were included. The random effect model (Mantel-Haenszel method) was applied. Abbreviation: CI confidence interval.

Twelve articles evaluated the effects of 5-HT_3_ receptor antagonists on global IBS symptom improvement [9, 20–25, 27, 30, 36–38]: six studies assessed ramosetron [9, 20–24], three studies investigated alosetron [25, 27, 30], and three studies compared cilansetron with placebo [36–38]. There was global IBS symptom improvement in 1990 (51%) of 3881 patients who received 5-HT_3_ receptor antagonists in comparison to 939 (33%) of 2865 patients receiving placebo or mebeverine; heterogeneity was statistically significant (*I*^*2*^ = 50%; 95% CI: 2.92, 74.20, *P* = 0.02) (Fig 2). The pooled RR from pooling effect size using a random effect model showed that 5-HT_3_ receptor antagonists improved global symptoms of IBS (RR = 1.56; 95% CI: 1.43, 1.71). Subgroup analysis showed that ramosetron improved global symptoms of IBS (RR = 1.49, 95% CI: 1.21, 1.83) with the same efficacy as alosetron (RR = 1.58, 95% CI: 1.42, 1.75) and cilansetron (RR = 1.66, 95% CI: 1.44, 1.90]) when compared with the control groups. Funnel plot asymmetry was not statistically significant (*P* = 0.903, Begg test), suggesting no evidence of publication bias or other minor study effects.

As heterogeneity was statistically significant, we conducted subgroup analysis, wherein heterogeneity was more obvious in the ramosetron group (*I*^*2*^ = 68%; 95% CI: 24.3, 86.5). We conducted sensitivity analysis to find the source of heterogeneity and to evaluate the robustness of the results, and found that the study by Lee et al. markedly affected heterogeneity [22]. The study involved 343 patients with IBS and had a Jadad score of 3. The authors indicated that both ramosetron and mebeverine were effective for improving global IBS symptoms, but this was not statistically significant. Removing this study significantly decreased heterogeneity; *I*^*2*^ decreased to 13%. The treatment effect appeared quite similar for both sexes (men and women: RR = 1.57 [95% CI: 1.33, 1.85] vs. women only: RR = 1.56 [95% CI: 1.43, 1.71]). Treatment durations of 24/48 weeks had lower RR (RR = 1.47 [95% CI: 1.26, 1.71]) compared to 4/12-week treatment (RR = 1.57 [95% CI: 1.42, 1.74]). Full papers had a lower RR (RR = 1.53 [95% CI: 1.35, 1.74]) than studies published as abstracts only (RR = 1.62 [95% CI: 1.43, 1.84]) (Table 2). We also analyzed the treatment effect subgroup for ramosetron according to dose and sex. The ramosetron doses used were 1, 1.25, 2.5, 5, or 10 μg once daily, and the duration of therapy was 4–12 weeks. However, when 2.5 μg once daily ramosetron was used for women and 5 μg once daily ramosetron was used for men, there was a trend toward higher efficacy in patients with IBS-D (2.5 μg: RR = 1.53 [95% CI: 1.28, 1.83]; 5 μg: RR = 1.52 [95% CI: 1.16, 2.01]), although this may have been because of a type II error, as the trials included fewer women, and the difference was not statistically significant (*P* = 0.89).

**Table 2:** Subgroup analyses of the efficacy endpoints.

Endpoint: global improvement of IBS symptoms	Subgroup	Studies (no.)	Subjects with IBS (no.)	RR of IBS symptoms improving	95% CI
Drug	Ramosetron	6	2552	1.49	1.21, 1.83
	Cilansetron	3	2230	1.66	1.44, 1.90
	Alosetron	3	1964	1.58	1.42, 1.75
Sex	Women only	5	2949	1.56	1.43, 1.71
	Mixed	7	3797	1.57	1.33, 1.85
Treatment duration	4/12 weeks	11	5954	1.57	1.42, 1.74
	24/48 weeks	1	792	1.47	1.26, 1.71
Publication type	Full paper	8	4107	1.53	1.35, 1.74
	Abstract only	4	2639	1.62	1.43, 1.84

**Fig 3:**
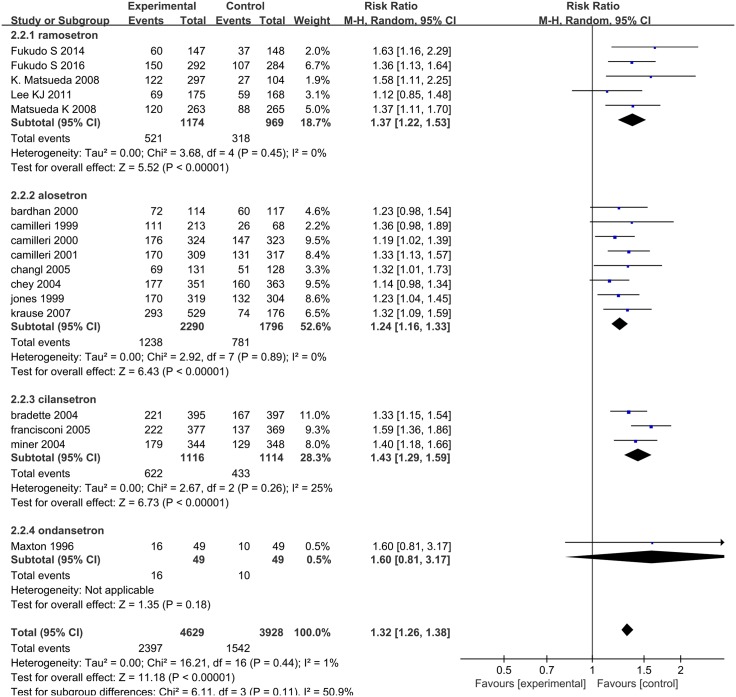
Forest plot of the relief of abdominal pain and discomfort. Seventeen articles were included. The random effect model (Mantel-Haenszel method) was applied. Abbreviation: CI confidence interval.

Seventeen articles evaluated the effects of 5-HT_3_ receptor antagonists on relief of abdominal pain and discomfort [9, 21–26, 28–29,31–34, 36–38]: five studies assessed ramosetron [9, 21–24], eight studies investigated alosetron [25–26, 28–29,31–34], three studies compared cilansetron with placebo [36–38], and one study investigated ondansetron [35]. Abdominal pain and discomfort were relieved in 2397 (52%) of 4629 patients assigned 5-HT_3_ receptor antagonists compared with 1542 (39%) of 3928 patients who received placebo or mebeverine; heterogeneity was not statistically significant (*I*^*2*^ = 1%; 95% CI: 0.00, 51.72, *P* = 0.44, Fig 3). The pooled RR for relief of abdominal pain and discomfort was 1.32 (95% CI: 1.26, 1.38). Subgroup analysis showed that ramosetron improved abdominal discomfort and pain (RR = 1.37, 95% CI: 1.22, 1.53) with the same efficacy as alosetron (RR = 1.24, 95% CI: 1.16, 1.33) and cilansetron (RR = 1.43, 95% CI: 1.29, 1.59) when compared with the control groups; ondansetron did not improve abdominal discomfort and pain with a statistically significant difference as compared with the controls (RR = 1.60, 95% CI: 0.81, 3.17). Funnel plot asymmetry was not statistically significant (*P* = 0.255, Begg test), suggesting no evidence of publication bias or other minor study effects.

**Fig 4:**
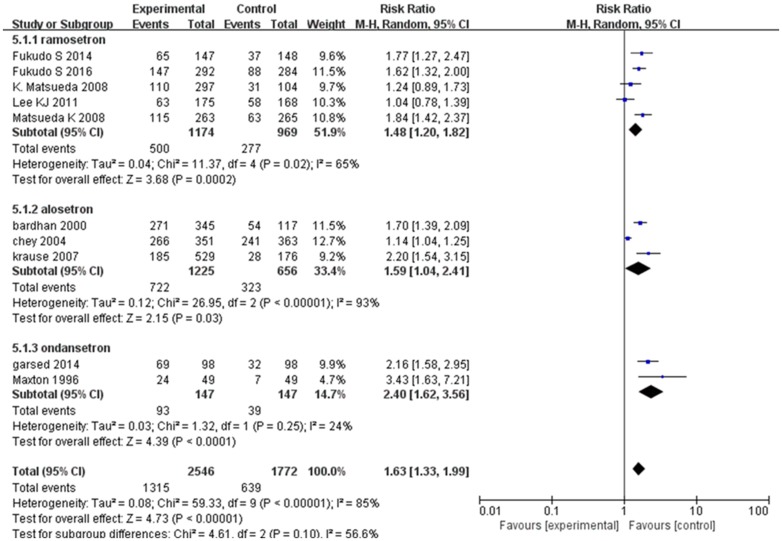
Forest plot of the improvement in abnormal bowel habits or stool consistency. Nineteen articles were included. The random effect model (Mantel-Haenszel method) was applied. Abbreviation: CI confidence interval.

Abnormal bowel habits or stool consistency were improved in 1315 (52%) of 2546 patients assigned 5-HT_3_ receptor antagonists compared with 639 (36%) of 1772 patients who received placebo or mebeverine. We used a random effect model (*I*^*2*^ = 85%; 95% CI: 73.81, 91.21) for the 10 RCTs on the effect of 5-HT_3_ receptor antagonists on abnormal bowel habits or stool consistency, finding that RR = 1.63 (95% CI 1.33–1.99) (Fig 4). Funnel plot asymmetry was statistically significant (Egger test, *P* = 0.025), suggesting publication bias or other minor study effects. Sensitivity analysis for determining the source of heterogeneity and for evaluating the robustness of the results determined that the studies of Chey et al. [28] (*n* = 714, Jadad score = 4) and Lee et al. [22] (*n* = 343, Jadad score = 3) markedly affected heterogeneity. In the former, stool consistency was defined as a GI symptom and the endpoint definition used a “Yes/No” response, while other studies used the US FDA definition [28]. Lee et al. indicated that both ramosetron and mebeverine were effective for improving abnormal bowel habits or stool consistency, but this was not statistically significant [22]. Removing the two studies significantly decreased heterogeneity; *I*^*2*^ decreased to 38%.

Subgroup analysis showed that ramosetron improved abnormal bowel habits or stool consistency (RR = 1.48, 95% CI: 1.20, 1.82) with the same efficacy as alosetron (RR = 1.59, 95% CI: 1.04, 2.41) and ondansetron (RR = 2.40, 95% CI: 1.62, 3.56) when compared with the control groups. The effect of 5-HT_3_ receptor antagonist treatment appeared quite similar in non-constipated IBS and IBS-D (non-constipated IBS and IBS-D: RR = 1.64 [95% CI: 1.08, 2.50] vs. IBS-D only: RR = 1.64 [95% CI: 1.35, 1.99]). Treatment durations of 24/48 weeks had lower RR (RR = 1.15 [95% CI: 1.00, 1.32]) compared to 4/12-week treatment (RR = 1.69 [95% CI: 1.43, 2.00]). The RR of the treatment effect was lower for women compared to men (men and women: RR = 1.66 [95% CI: 1.34, 2.05] vs. women only 1.56 [95% CI: 1.08, 2.24]) (Table 3).

**Table 3:** Subgroup analyses of the efficacy endpoints.

Endpoint: improvement of abnormal bowel habits or stool consistency	Subgroup	Studies (no.)	Subjects with IBS (no.)	RR of IBS symptoms improving	95% CI
Drugs	Ramosetron	5	2143	1.48	1.20, 1.82
	Ondansetron	2	294	2.4	1.62, 3.56
	Alosetron	3	1881	1.59	1.04, 2.41
Sex	Women only	3	1995	1.56	1.08, 2.24
	Mixed	7	2323	1.66	1.34, 2.05
Treatment duration	4–12 weeks	9	3604	1.69	1.43, 2.00
	48 weeks	1	714	1.15	1.00, 1.32
Study population	Mixed (non-constipated and IBS-D)	3	1274	1.64	1.08, 2.50
	IBS-D only	7	3044	1.64	1.35, 1.99

### Adverse events following administration of 5-HT_3_ receptor antagonist for treating IBS

**Fig 5:**
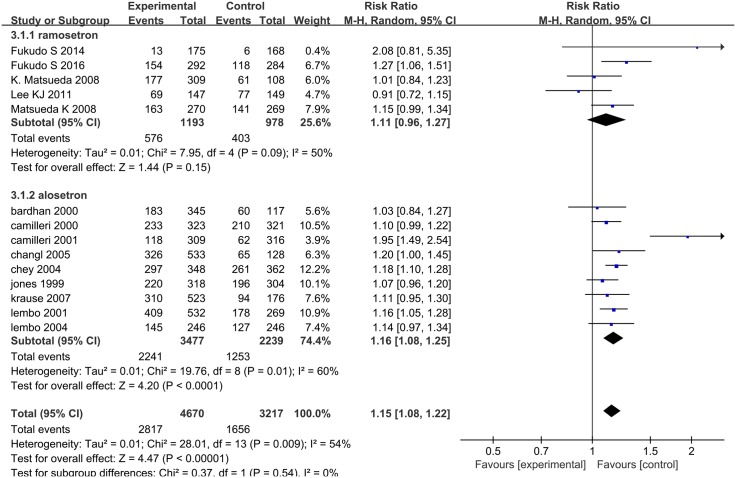
Forest plot of the rate of adverse effects. Fourteen articles were included. The random effect model (Mantel-Haenszel method) was applied. Abbreviation: CI confidence interval.

Fourteen RCTs evaluated the rate of adverse effects: five were on ramosetron [9, 21–24] and nine were on alosetron [25–32, 34], and involved 7887 patients in total. Heterogeneity was statistically significant (*I*^*2*^ = 54%; 95% CI: 14.96, 74.67, *P* = 0.009, Fig 5). We used a random effect model to pool effect size, where the pooled RR of the rate of adverse events was 1.15 (95% CI: 1.08, 1.22). Adverse events were not statistically significant in the ramosetron group (RR = 1.11, 95% CI: 0.96, 1.27). In the alosetron group, the pooled RR of the rate of adverse events was 1.16 (95% CI: 1.08, 1.25). The cilansetron and ondansetron studies did not report adverse events data. The alosetron treatment group recorded nine ischemic colitis cases; the control group had none. Funnel plot asymmetry was not statistically significant (*P* = 0.870, Begg test), suggesting no evidence of publication bias or other minor study effects.

**Fig 6:**
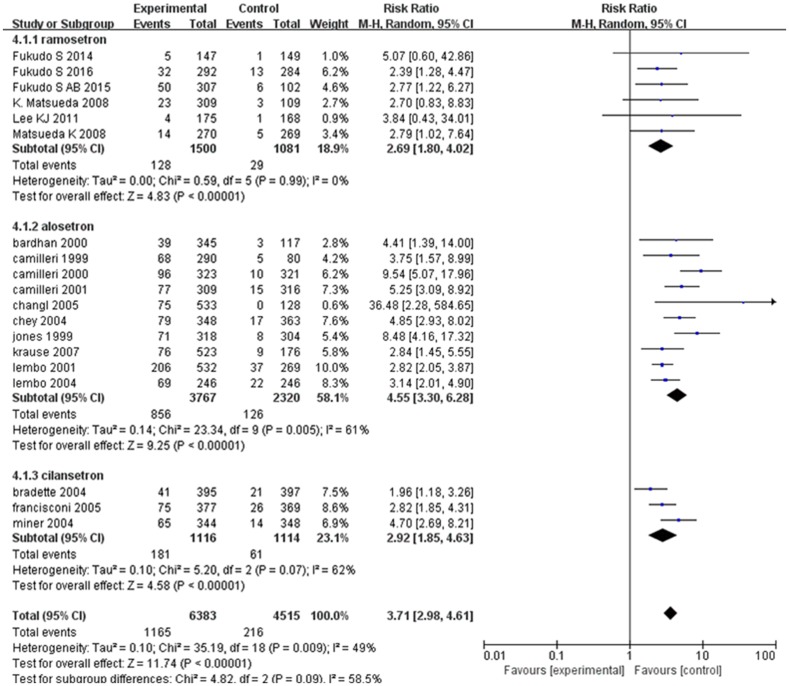
Forest plot of the rate of developing constipation. Ten articles were included. The random effect model (Mantel-Haenszel method) was applied. Abbreviation: CI confidence interval.

We analyzed the rate of constipation to analyze the effect of 5-HT_3_ receptor antagonists on IBS accurately, which involved 10,898 patients and determined that heterogeneity was statistically significant (*I*^*2*^ = 49%; 95% CI: 12.99, 69.93, *P* = 0.009, Fig 6). We used a random effect model to pool effect size, where the pooled RR showed that the 5-HT_3_ receptor antagonist treatment group had a higher rate of constipation (RR = 3.71, 95% CI: 2.98, 4.61) than the control group. One hundred and twenty-eight patients (9%) who received ramosetron reported constipation compared with 29 patients (3%) in the placebo arm (RR = 2.69; 95% CI: 1.80, 4.02) [9, 20–24]. Eight hundred and fifty-six patients (23%) who received alosetron reported constipation compared with 126 patients (5%) in the placebo arm (RR = 4.55; 95% CI: 3.30, 6.28) [25–34]. One hundred and eighty-one patients (16%) who that received cilansetron reported constipation compared with 61 patients (5%) in the placebo arm (RR = 2.92; 95% CI: 1.85, 4.63) [36–38]. Patients with non-constipated IBS may develop constipation more often than patients with IBS-D (non-constipated IBS and IBS-D: RR = 5.28 [95% CI: 3.93, 7.08] vs. IBS-D only: RR = 3.24 [95% CI: 2.54, 4.12]), and the RR of the rate of constipation was lower in men compared to women (men and women: RR = 3.13 [95% CI: 2.37, 4.12] vs. women only: RR = 4.05 [95% CI: 2.96, 5.54])(Table 4).

**Table 4:** Subgroup analyses of the efficacy endpoints.

Endpoint: Number of patients developing constipation	Subgroup	Studies (no.)	Subjects with IBS (no.)	RR of IBS symptoms improving	95% CI
Drugs	Ramosetron	6	2581	2.69	1.80, 4.02
	Alosetron	10	6087	4.55	3.30, 6.28
	Cilansetron	3	2230	2.92	1.85, 4.63
Sex	Women only	9	5579	4.05	2.96, 5.54
	Mixed	10	5319	3.13	2.37, 4.12
Treatment duration	4/12 weeks	17	9359	3.13	2.37, 4.12
	24/48 weeks	2	1503	3.09	1.27, 7.52
Study population	Mixed (non-constipated and IBS-D)	5	2790	5.28	3.93, 7.08
	IBS-D only	14	8180	3.24	2.54, 4.12
Publication type	Full paper	15	8259	4.08	3.14, 5.30
	Abstract only	4	2639	2.89	2.01, 4.15

## Discussion

The present meta-analysis indicates significant improvement of the global symptoms, abdominal pain and discomfort, and abnormal bowel habits or stool consistency in non-constipated IBS or IBS-D following 5-HT_3_ receptor antagonist treatment. Our results are consistent with the systematic reviews by Ford et al. and Andresen et al. [4, 15], who concluded that alosetron and cilansetron improve global IBS symptoms and abdominal pain. Previous systematic reviews and meta-analyses that examined alosetron and cilansetron efficacy for treating IBS have important limitations. Andresen et al. included minor inconsistent data in their meta-analysis, which included the application of the proportion of patients that improved after therapy from a subgroup analysis, rather than an intention-to-treat population, to calculate the number of patients whose symptoms improved [15]. Ford et al. provided efficacy data for all existing 5-HT receptor agonists and antagonists used for treating IBS and concluded that alosetron, cilansetron, and tegaserod were all more effective than placebo for treating IBS [4]. Unfortunately, Ford et al. and Andresen et al. did not include studies on ramosetron or ondansetron in their analyses, or the US FDA-suggested use of stool consistency and abdominal pain as co-primary endpoints for IBS-D. The quality of the RCTs included often limit meta-analyses. In the present study, trial quality, assessed using the Jadad scale, fortunately was high for most of the studies. We used intention-to-treat analysis and used a random effects model to pool data to obtain more conservative estimates of 5-HT_3_ receptor antagonist efficacy, and contacted the authors to obtain supplementary data to maximize the number of potentially eligible trials that could contribute data to our analyses. Moreover, we assessed the potential adverse effects of these therapies by collecting and pooling adverse events data. Lastly, we used subgroup analyses to examine the effect of different 5-HT_3_ receptor antagonists, sex and duration of therapy, study population, and publication type. We found consistent treatment response across studies from different countries; for “relief of global IBS symptoms”, “relief of abdominal pain and discomfort” and “relief of abnormal bowel habits or stool consistency”, the estimated pooled RR was 1.56 (95% CI: 1.43, 1.71), 1.33 (95% CI: 1.26, 1.39), and 1.63 (95% CI: 1.33, 1.99), respectively.

For the endpoint “relief of global IBS symptoms”, the six ramosetron studies had statistically significant heterogeneity [9, 20–24]: five analyzed the effect of ramosetron compared with placebo, indicating that ramosetron improves global IBS symptoms [9, 20, 21, 23, 24]. Only one study used mebeverine as compared with ramosetron and indicated that both ramosetron and mebeverine were effective, but the treatment groups did not differ statistically significantly from the control groups [22]. However, heterogeneity was significant in that study, and its Jadad score was one of the two lowest among all studies and was lower than that of the other ramosetron studies. Removing this study significantly decreased heterogeneity. Our analysis suggests that there are more obvious beneficial effects following ramosetron treatment. However, subgroup inferences were confounded because all studies on cilansetron involved both patients of both sexes and were only available as abstracts [36–38]. Consequently, we could not draw conclusions on the efficacy of cilansetron in male or female patients. Treatment duration was another significant subgroup–treatment interaction that influenced the RR for this outcome, where the RR of long-term treatment was lower, suggesting that the effect of treatment might abate over time. However, only one study involved long-term treatment (24–48 weeks) compared to 11 studies with 4–12-week treatment, which does not permit a definite conclusion as to whether treatment efficacy wanes with time. Alosetron has been approved in the United States only for female patients; in contrast, the clinical efficacy of ramosetron for IBS-D has been shown only in men [8]. These data resulted in ramosetron being limited to male patients with IBS-D in Japan, Korea, and Thailand. In subgroup analysis of the endpoint “relief of global IBS symptoms” for ramosetron, which included four studies each on IBS-D in male and female patients, we found that ramosetron has similar efficacy for both male and female patients with IBS-D (men and women: RR = 1.90 [95% CI: 1.50, 2.40] vs. women only: RR = 1.85 [95% CI: 1.44, 2.39]). We also performed subgroup analyses to examine the effect of ramosetron dose and sex on the improvement of global IBS symptoms. The analyses showed that ramosetron was less effective in women, although this difference was not statistically significant. Fukudo et al. indicated that 2.5 μg/day ramosetron is an effective treatment for IBS-D in women [9]. In contrast, Matsueda et al. and Fukudo et al. discovered the optimal ramosetron dose for male patients is 5 μg/day [21–24]. Several factors have been suggested for these differences [39]. They include significant retardation by alosetron of small intestinal and colonic transit in women with IBS-D as compared with men, i.e., sex partly contributes to differences in the serotonergic control of intestinal and colonic transit in patients with D-IBS [40], more systemic exposure to alosetron was associated with inhibition of cytochrome P450 CYP1A2 in women than in men [41], and brain responses to visceral stimulation by alosetron in IBS are based on sex [42]. The activation of brain networks involved with cognitive, autonomic, and antinociceptive responses to delivered and anticipated aversive visceral stimuli differ in male and female patients with IBS [43]. Whatever the mechanisms, women with IBS-D need half the ramosetron dose as men with IBS-D do.

The assessments of the effects of 5-HT_3_ receptor antagonists on “relief of abdominal pain and discomfort” and “relief of global IBS symptoms” are relatively common measurements. This is the first time our study accepted the outcome “relief of abnormal bowel habits or stool consistency” as an endpoint for evaluating 5-HT_3_ receptor antagonist efficacy for treating IBS. We included two studies on ondansetron that only included the endpoint “abnormal bowel habits or stool consistency improving” [12, 35]; previous studies have not included this drug. However, the included studies used inconsistent assessment criteria. Consequently, we included a limited number of studies evaluating both symptoms according to our strict meta-analysis criteria. There might have been inadequate power for detecting significant differences due to this data scarcity, which may explain the higher efficacy of cilansetron on “relief of abdominal pain and discomfort” and “relief of abnormal bowel habits or stool consistency” as compared to the other groups. While global IBS symptoms is a general IBS symptom, abdominal discomfort, the sense of bloating, stool consistency, and change in bowel habits all likely affect patients’ overall experiences. Therefore, separate evaluation of abdominal pain and abnormal bowel habit improvements highlighted interferences resulting from representative symptoms of IBS and was more reasonable. The subgroup analyses examined the effect of treatment, sex and duration of therapy, study population, and publication type, and showed that 5-HT_3_ receptor antagonists are less effective in women, although this difference is opposite to the endpoint “relief of global IBS symptoms”. These analyses showed that 5-HT_3_ receptor antagonists are more effective in IBS-D only.

5-HT_3_ receptor antagonists are considered safe for treating non-constipated IBS or IBS-D. The placebo-controlled studies reported few serious adverse events [44]. The pooled RR between the ramosetron and control groups did not differ statistically significantly. The adverse events reported between the alosetron and control groups often differed statistically significantly. However, these 5-HT_3_ receptor antagonists were either withdrawn from the market (alosetron) or never marketed (cilansetron) due to concerns regarding the complication of severe constipation and reports of ischemic colitis. Garsed et al. showed that ondansetron can achieve useful results with a low incidence of adverse effects. While their small study cannot prove the safety of ondansetron, the fact that it has been used widely for over 25 years without a single report of ischemic colitis suggests that this adverse effect is rare [12]. For the outcome rate of constipation, ramosetron had a lower pooled RR than the other comparisons between treatment and control groups. There was no ischemic colitis in 1418 patients with IBS-D who received ramosetron. Moreover, the incidence of constipation was much lower in ramosetron-treated patients than in patients treated with alosetron or cilansetron. Finally, ramosetron is associated with a low incidence of adverse events, such as abdominal distension and hard stool, and is unlikely to cause ischemic colitis [45]. Based on the above analysis, ramosetron is safe for treating non-constipated IBS or IBS-D. However, clinical research has been carried on this drug only in Japan and Korea. Consequently, these findings may not be generalizable to Western populations. There was rate of constipation in the 5-HT_3_ receptor antagonist treatment group was 18%. There was lower constipation risk in trials that included only patients with IBS-D as compared to studies that included patients with both non-constipated IBS and IBS-D, indicating that patients with IBS-D may have a more favorable benefit risk ratio for 5-HT_3_ receptor antagonist treatment [46]. Ondansetron benefits in IBS-D were observed mainly in terms of improvement in stool consistency and urgency as well as increased gut transit time. These effects occurred rapidly within 7 days; <10% of patients had constipation and there was no ischemic colitis. These findings have important implications for clinicians, as ondansetron is inexpensive and with a good safety profile [12].

### Limitations and strengths

The limitations and strengths of our systematic review relate to the primary data and to the review itself. Four studies (one on ramosetron and three on cilansetron) were only available as abstracts; therefore, we could not fully evaluate their methodological quality. Nevertheless, 16 of the included RCTs were high-quality, full-text trials (Jadad score = 4). Another strength of the primary data is that the endpoints were comparable and standardized and all studies had similar trial designs. One limitation was that we could not exclude publication bias and reporting bias. Detection and accurate estimation of heterogeneity are very important in a meta-analyses study. Whenever heterogeneity is detected, it should not be ignored. A zero between-study variance may provide a reliable result, while high levels of estimated heterogeneity may potentially have an effect on combination of the separate estimate into a single result. In this study, we used random effect models for all the endpoints “relief of global IBS symptoms”, “relief of abdominal pain and discomfort”, “relief of abnormal bowel habits or stool consistency”, and “adverse event rate”. Additionally, our subgroup analyses and sensitivity analyses were used to find the source of heterogeneity as far as possible, there may still have unobserved heterogeneity. We have not checked the sensitivity of the meta-analysis conclusion to assumed moderate and large degrees of heterogeneity, which is a limitation in our present study. Another limitation is that we calculated the estimated RR based on published papers and abstracts, particularly those on cilansetron; consequently, the effects of ramosetron should be interpreted with caution with regard to the apparent inter-study heterogeneity and low number of included studies.

The main strength of this review is its comprehensive approach. First, we included six and two studies on ramosetron and ondansetron, respectively. Ondansetron is a classic 5-HT_3_ receptor antagonist widely used as an antiemetic that, in small trials, has benefited patients with IBS. Recently, a large RCT involving 120 patients with IBS-D showed that ondansetron may be efficacious against IBS. Previous meta-analyses did not analyze ramosetron and ondansetron for treating IBS; we are the first to have done so. Second, we evaluated the effects of 5-HT_3_ receptor antagonists according to abnormal bowel habits or stool consistency. With this method, we were able to assess the effects of 5-HT_3_ receptor antagonists on bowel habits and stool consistency rather than limiting the symptom profile to abdominal pain. Furthermore, these fixed criteria also avoid the random assessment of relief of abnormal bowel habits or stool consistency and therefore avoid overestimating the effects of 5-HT_3_ receptor antagonists. Third, the previously published meta-analyses rarely referred to adverse events. Our meta-analysis evaluated 5-HT_3_ receptor antagonist safety based on the adverse effects, and analyzed the pooled rate of constipation. Fourth, we included four studies only available as abstracts. Excluding these studies would have increased random errors and publication bias, as these were large, multi-center trials involving >2500 patients, were of high randomization and blinding quality, and defined the study populations and outcomes sufficiently. Moreover, these studies reported the only available data evaluating cilansetron and ramosetron efficacy and safety in large, multi-center trials on IBS. Including studies on both ramosetron and ondansetron allowed us to combine all available RCTs on 5-HT_3_ receptor antagonists for treating non-constipated IBS with regard to the main clinical endpoints, strengthening the validity of our results on the effects of this class of drugs for treating non-constipated IBS.

## Conclusions

This systematic review and meta-analysis finds that 5-HT_3_ receptor antagonists improve global IBS symptoms, abdominal pain, and abnormal bowel habits or stool consistency in non-constipated IBS and IBS-D. Constipation is a common, but usually mild to moderate adverse effect of 5-HT_3_ receptor antagonist treatment. The risk of constipation is lower in IBS-D and this underscores the importance of assessing the individual benefit/risk ratio before starting treatment. Ischemic colitis is a rare adverse event with ramosetron. Ischemic colitis and constipation remain of concern to the regulatory agencies and have led to cilansetron and alosetron being restricted to patients with severe refractory IBS-D who have failed to respond to conventional treatment.

## Supporting information

S1 Text PRISMA 2009 checklist. (DOC)

S2 Text Certification. (PDF)
