# Supplementary material for: Efficacy and safety of 5-hydroxytryptamine 3 receptor antagonists in irritable bowel syndrome: A systematic review and meta-analysis of randomized controlled trials
Source: PLoS One. 2017 Mar 14;12(3):e0172846. doi: 10.1371/journal.pone.0172846 (PMC5349445; doi:10.1371/journal.pone.0172846)
Supplement: S2 Text — (PDF) [file pone.0172846.s002.pdf]

www.elixigen.com

Editorial Certification

This document certifies that the manuscript titled "Efficacy and Safety of 5-Hydroxytryptamine 3 Receptor Antagonists in Irritable Bowel Syndrome: A Systematic Review and Meta-analysis of Randomized Controlled Trials" was edited for proper English language, grammar, punctuation, spelling, and overall style by one or more of the highly qualified native English speaking editors at ELIXIGEN.

Neither the research content nor the authors' intentions were altered in any way during the editing process.

Documents receiving this certification should be English-ready for publication - however, the author has the ability to accept or reject our suggestions and changes. To verify the final ELIXIGEN edited version, please contact ELIXIGEN at support@elixigen.com
